# Safety and patient-reported outcomes in index ablation versus repeat ablation in atrial fibrillation: insights from the German Ablation Registry

**DOI:** 10.1007/s00392-020-01763-1

**Published:** 2020-10-28

**Authors:** Shinwan Kany, Johannes Brachmann, Thorsten Lewalter, Karl-Heinz Kuck, Dietrich Andresen, Stephan Willems, Ellen Hoffmann, Lars Eckardt, Dierk Thomas, Matthias Hochadel, Jochen Senges, Andreas Metzner, Andreas Rillig

**Affiliations:** 1 Department of Cardiology, University Heart and Vascular Center Hamburg-Eppendorf, Martinistraße 52, 20251 Hamburg, Germany; 2 Department of Cardiology, Angiology and Pneumology, Coburg Hospital, Coburg, Germany; 3 Department of Medicine-Cardiology and Intensive Care, Hospital Munich-Thalkirchen, Munich, Germany; 4 LANS Cardiology, Hamburg, Germany; 5 Department of Cardiology, Evangelisches Krankenhaus Hubertus, Berlin, Germany; 6 Department of Cardiology, Asklepios Klinik St. Georg, Hamburg, Germany; 7 Dept. of Cardiology and Internal Intensive Care Medicine, Heart Center Munich-Bogenhausen, Munich Clinic Bogenhausen, Munich, Germany; 8 Department of Cardiology (Electrophysiology), University Hospital Muenster, Muenster, Germany; 9 Department of Cardiology, University of Heidelberg, Heidelberg, Germany; 10 Stiftung Für Herzinfarktforschung (IHF), Ludwigshafen, Germany

**Keywords:** Atrial fibrillation, Repeat ablation, Patient-reported outcomes

## Abstract

**Background:**

Pulmonary vein isolation is an established strategy for catheter ablation of atrial fibrillation (AF). However, in a significant number of patients, a repeat procedure is mandatory due to arrhythmia recurrence. In this study, we report safety data and procedural details of patients undergoing index ablation versus repeat ablation in a registry-based real-life setting.

**Methods:**

Patients from the German Ablation Registry, a prospective, multicentre registry of patients undergoing ablation between January 2007 and January 2010 were included.

**Results:**

A total of 4155 patients were enrolled in the study. Group I (index ablation) consisted of 3377/4155 (82.1%) and group II (repeat ablation) of 738/4155 (17.9%). Patients in group I had a significantly higher ratio of paroxysmal AF (69.3% vs 61.9%, *p* < 0.001) and significantly less persistent AF (30.7% vs 38.1%, *p* < 0.001). The repeat group showed significantly lower mean RF application duration (2580 s. vs 1960, *p* < 0.001), less fluoroscopy time (29 min. vs. 27 min., *p* < 0.001), less mean dose area product (DAP) (3744 cGy × cm^2^ vs 3325 cGy × cm^2^, *p* = 0.001), and shorter study duration (181.2 min. vs 163.6 min., *p* < 0.001). No statistical difference between the groups was found in terms of mortality (0.3% vs 0.1%, *p* = 0.39), MACE (0.4% vs 0.3%, *p* = 0.58), MACCE (0.8% vs 0.6%, *p* = 0.47), composite safety endpoint (1.5% vs 1.4%, *p* = 0.76), and arrhythmia recurrence (43.8% vs 41.9%, *p* = 0.37) during 1-year follow-up. Both groups reported to have improved or no symptoms (80.4% vs 77.8%, *p* = 0.13).

**Conclusion:**

Repeat catheter ablation is safe and provides a symptomatic relief comparable to index ablation. Repeat procedures are significantly shorter and use less fluoroscopy.

## Introduction

Atrial fibrillation (AF) is the most common arrhythmia affecting humans and is, in addition to oftentimes typical symptoms and reduced quality of life, associated with a rise of associated mortality [1]. According to the most recent guidelines, catheter ablation via pulmonary vein isolation (PVI) is a class I recommendation for patients with either paroxysmal or persistent atrial fibrillation after failed antiarrhythmic drug therapy [2]. Recently, randomized-controlled trials comparing radiofrequency (RF) and cryoballoon (CB) ablation in AF showed that either method is associated with high freedom of arrhythmias [3–5]. A significant portion of patients is in need of a repeat ablation due to re-connection of previously isolated pulmonary veins [4]. Currently, more progressive forms like persistent AF and long-standing persistent AF show a greater tendency to require more than one ablation to increase the probability of long-lasting success [4]. No ablation strategy besides PVI has been proven to comprehensively and reproducibly reduce the recurrence of AF after index ablation. Additionally, the 2020 ESC guidelines recommend collecting patient-reported outcomes (PRO) to assess success of patients care [2]. Furthermore, repeated catheter ablation is given a class IIa recommendation if the index procedure provided symptomatic relief [2].

Large real-world data comparing repeat vs. index ablation and differential ablation strategies remain scarce. Additionally, the patient cohort undergoing repeat ablation as well as procedural safety and efficacy have only been characterized in select populations.

This study aims to analyze repeat procedures compared to index procedures regarding procedural details as well as safety and PRO such as symptomatic burden and satisfaction in a prospective, multicentre registry.

## Methods

### German ablation registry structure

The non-profit organization “Institut für Herzinfarktforschung” (IHF, Ludwigshafen, Germany) is supervising the prospective, multi-center German Ablation Registry. Out of 55 participating German centers, 41 provided cases with AF catheter ablation.

### Patient cohort

Patients with age > 18 years undergoing catheter ablation for symptomatic AF between January 2007 and January 2010 at participating centers were enrolled. Patients with AV node ablation or with long-standing persistent AF were excluded from the analysis. The cohort was divided into two groups undergoing either index (group I) or repeat ablation (group II) and compared. Written consent for catheter ablation and participation for the registry were obtained beforehand. The ethics committee of the Rhineland‐Palatinate State Medical Council (Landesärztekammer Rheinland‐Pfalz) approved the study (No. 837.026.07 (5561)).

### Procedural methods for catheter ablation

Procedures were performed in accordance with standard protocols at participating centers. Procedure protocols have been described in detail before [6–8]. Transthoracic echocardiography was performed to assess left-ventricular ejection fraction (LVEF) and left atrial (LA) diameter. To rule out intracardiac thrombi, transesophageal echocardiography was performed. Additional pre-procedural imaging was left to each participating partner. Procedures were conducted under deep sedation. Hemodynamic parameters including, but not limited to, blood pressure, heart rate, and oxygen saturation were monitored.

An activated clotting time (ACT) target of 300–400 s was maintained throughout the procedure. Ablation and transseptal access were guided by fluoroscopy during CB and RF. Only the first-generation CB (Medtronic, Minneapolis, MN, USA) was available for the procedures. In the latter case, electroanatomical mapping (EAM) systems such as CARTO or NavX were used according to the operator’s preference. PVI was confirmed using diagnostic catheters. Additional ablation strategies may include linear lesions of either right atrium (RA) or LA such as the cavo-tricuspid isthmus (CTI) area, as well as ablation of complex fractionated atrial electrograms (CFAEs), and were left to the operator’s choice. During CB ablation, continuous monitoring of the phrenic nerve was achieved via fluoroscopy or pacing maneuvers. After the procedure, pressure bandages or figure of eight sutures were applied to the groin area. Anti-arrhythmic drug therapy (AAD) and post-procedural anticoagulation management were left to standard procedures at participating sites.

### Registry management and clinical follow-up

The IHF is managing project development, data acquisition, and clinical monitoring. Physicians and study nurses at participating sites entered data for baseline characteristics, procedural details, and 30-day follow-up. Any arrhythmia was documented via ECG tracings and/or Holter recording. The IHF conducted a 1-year follow-up using electronic health records and telephone calls for standardized questionnaires. In between this time-period, clinical follow-up occurred at each participating center’s discretion including (but not mandatory) Holter monitoring. For assessment of AF symptomatic burden, the severity of AF scale (SAF) was used. Electronic data management with Internet-based care report forms (EBogen©, developed by the IHF) using secure encryption was employed. Statistical analysis and biomedical models were carried out by the IHF.

### Outcomes

The primary outcome was the acute procedural success and safety. Major adverse cardiac event (MACE) was defined as death or myocardial infarction (MI). Major adverse cardiac and cerebrovascular events (MACCE) was defined as death, MI, or stroke. Composite safety endpoint was defined as death, MI, stroke, or major bleeding.

Secondary endpoints were patient survival, long-term procedural success, and safety. Additionally, patient-oriented outcome such as quality of life and satisfaction with treatment were considered secondary endpoints.

Potential periprocedural complications were categorized into severe, moderate, and minor complications.

### Statistical analysis

Normally distributed continuous data are shown as means ± standard deviation (SD), otherwise given as medians with first and third quartiles. Categorical data are shown in relative percentages and absolute values. Statistical differences between both groups were compared using either Mann–Whitney–Wilcoxon test or with a Chi-square test. For rates of in-hospital complications, Fisher’s exact test was used. The 12-month event-rates of MACCE (composite outcome of death, myocardial infarction, and stroke), MACE (composite outcome of death and myocardial infarction), and composite safety endpoint (composite endpoint of death, myocardial infarction, stroke, and major bleeding) were calculated by the Kaplan–Meier method. The aforementioned outcomes were compared between age groups using the log-rank test. All statistical comparisons were two-sided, and *P *values < 0.05 were considered statistically significant. Analyses were performed using the Statistical Analysis System (SAS, Version 9.4, SAS Institute Inc., Cary, NC, USA).

## Results

### Patient cohort and baseline parameters

A total of 4155 patients were included in the study. Group I (index ablation) consisted of 3377/4155 (82.1%) and group II (repeat ablation) of 738/4155 (17.9%). The proportion of female patients was higher in patients of the index cohort (33.5% vs 29.1%, *p *= 0.021), whereas median age was lower (62 vs 63 years; *p* = 0.021). Patients from group I compared to group II showed no statistically different comorbidities in diabetes mellitus (DM), chronic kidney disease (CKD), hypertension, and prior stroke as well as chronic obstructive pulmonary disease (COPD). Both groups had comparable proportions of prior device implantation like pacemaker (PM), implantable cardioverter defibrillator (ICD), or cardiac resynchronisation therapy (CRT). Group I and group II were similar with regards to comorbidities such as coronary artery disease (CAD), prior myocardial infarction, and cardiomyopathy. Patients undergoing index ablation were less likely to have valvular heart disease (6.6% vs 9.1%, *p* = 0.016). LVEF was preserved in either group at 86.8% vs 88.0% (*p* = 0.40). Group I had a significantly lower portion of patients with severe symptoms (New York Heart Association class, NYHA 2 +) compared to group II (39.7% vs 49.4%, *p* = 0.004). CHA_2_DS_2_-VASc Scores were comparable in both groups (1.8 ± 1.3 vs 1.7 ± 1.2, *p* = 0.63). Patients at index ablation (group I) had a significantly higher proportion of paroxysmal AF (69.3% vs 61.9%, *p* < 0.001) and significantly less persistent AF (30.7% vs 38.1%, *p* < 0.001). Patients with long-standing persistent AF were not included in the analysis. An overview of the traits of each group is given in Table 1.

**Table 1:** Baseline characteristics of both patients undergoing index versus repeat ablation

	Index (*n* = 3377)	Repeat (*n* = 738)	Odds ratio (95%-CI) or p-value
Age (years)	62 (54; 68)	63 (55; 68)	*P* = 0.69
Female sex	33.5%	29.1%	1.23 (1.03–1.46)
Diabetes mellitus	7.8%	6.8%	1.17 (0.86–1.60)
Chronic kidney disease	2.1%	2.8%	0.76 (0.21–2.72)
Hypertension	61.1%	64.5%	0.86 (0.56–1.32)
Stroke	5.6%	4.6%	1.24 (0.47–3.26)
COPD	1.2%	2.8%	0.44 (0.11–1.73)
Cardiac device (PM, ICD, CRT)	5.9%	6.2%	0.95 (0.68–1.32)
Pacemaker	4.4%	5.3%	1.20 (0.84 -1.72)
ICD	1.4%	0.9%	0.66 (0.30–1.47)
CRT	0.1%	0.1%	2.29 (0.21–25.28)
Coronary artery disease	18.2%	16.1%	1.15 (0.93–1.43)
Prior myocardial infarction	4.6%	6.2%	0.73 (0.52–1.03)
Cardiomyopathy (HCM, DCM)	3.9%	3.8%	1.04 (0.69–1.58)
Valvular heart disease	6.6%	9.1%	0.70 (0.53–0.94)
LVEF preserved (> 50%)	86.8%	88.0%	0.90 (0.69–1.17)
CHA_2_DS_2_-VASc Score	1.8 ± 1.3	1.7 ± 1.2	*P* = 0.63
NYHA 2 +	39.7%	49.4%	0.67 (0.51–0.88)
Atrial fibrillation type			
Paroxysmal	69.3%	61.9%	*P* < 0.001
Persistent	30.7%	38.1%	*P* < 0.001

All values given as percentages or mean with standard deviation or quartiles. *P* value < 0.05 was considered significant

*CI* confidence interval, *COPD* Chronic Obstructive Pulmonary Disease; *PM* Pacemaker; *ICD* Implantable Cardioverter Defibrillator; *CRT* Cardiac Resynchronization Therapy; *HCM* Hypertrophic Cardiomyopathy; *DCM* Dilatative Cardiomyopathy; *LVEF* Left-Ventricular Ejection Fraction; *NYHA* New York Heart Association

### Procedural details

Before ablation rhythm at ablation in both groups was comparable at sinus rhythm (SR) and AF. Compared to repeat ablation, PVI at index procedure was carried out significantly more often using a circumferential approach (87.9% vs 78.0%, *p* < 0.001) and less-segmental isolation (12.4% vs 16.0%, *p* = 0.008). Linear lesions were significantly less common in the index group compared to the repeat group (14.6% vs 26.6%, *p* < 0.001). Of those lesions, the index group demonstrated a lower proportion of linear lesions in the LA (49.2% vs 60.7%, *p* = 0.006), but no difference in RA lesions (56.7% vs 57.7%, *p* = 0.82) and CTI ablation (97.1% vs 92.9%, *p* = 0.055). CFAE ablation was conducted significantly less often in the index ablation group (7.8% vs 25.1%, *p* < 0.001). Both conventional mapping as well as EAM were utilized similarly between the groups. Pre-procedural imaging was statistically more prevalent at index ablation (25.2% vs 16.7%, *p* < 0.001), driven by computed tomography (CT) (18.9% vs 12.3%, *p* < 0.001). Group I was treated with significantly less RF energy (77% vs 94.6%, *p* < 0.001) and more CB ablation (21.7% vs 5.4%, *p* < 0.001). Acute procedural success was the same for both groups (96.3% vs 97.0%, *p* = 0.36). The index group showed significantly higher mean RF application duration (2580 s. vs 1960, *p* < 0.001), longer fluoroscopy time (29 min. 27 min., *p* < 0.001), more mean dose area product (DAP) (3744 cGy × cm^2^ vs 3325 cGy × cm^2^, *p* = 0.001), and longer study duration (181.2 min. vs 163.6 min., *p* < 0.001). An overview of procedural data is given in Table 2.

**Table 2:** Procedural details of AF patients undergoing index versus repeat ablation

	Index (*n* = 3377)	Repeat (*n* = 738)	*p* value
Energy form used			
Radiofrequency	77.0%	94.6%	< 0.001
Cryoballoon	21.7%	5.4%	< 0.001
Rhythm before ablation			
Sinus rhythm	68.5%	68.2%	0.85
Atrial fibrillation	31.5%	31.8%	0.85
Pulmonary vein isolation			
Circumferential	87.9%	78.0%	< 0.001
Segmental	12.4%	16.0%	0.008
Ablation strategy			
Linear lesion	14.6%	26.6%	< 0.001
Location LA	49.2%	60.7%	0.006
Location RA	56.7%	57.7%	0.82
Location CTI	97.1%	92.9%	0.055
CFAE	7.8%	25.1%	< 0.001
Irrigated-tip catheters	70.0%	90.7%	< 0.001
Mapping, imaging, and study			
Conventional mapping	39.3%	40.2%	0.63
EAM	58.0%	58.3%	0.88
Pre-procedural imaging	25.2%	16.7%	< 0.001
Mean RF application duration (sec)	2580 (1657; 3656)	1960 (1038; 3405)	< 0.001
Mean fluoroscopy time (min)	29 (20; 46)	27 (17; 44)	< 0.001
Mean dose area product (cGY × cm^2^)	3744 (1898; 7234)	3325 (1754; 6229)	0.001
Mean study duration (min)	181.2 ± 71.9	163.6 ± 67.1	< 0.001

All values are given as percentages or mean with standard deviation or quartiles. *P* value < 0.05 was considered significant

*LA* Left Atrium; *RA* Right Atrium; *CTI* Cavo-tricuspid Isthmus; *CFAE* Complex Fractioned Atrial Electrograms; *EAM* Electroanatomical Mapping; *RF* Radiofrequency; *sec* Seconds; *min* Minutes

### In-hospital complications and safety

There was one death in group I during hospital stay. Severe non-fatal adverse events (AE) and moderate non-fatal AE were comparable in both groups. Mild AE were significantly less for the repeat ablation cohort (3.8% vs 2.0%, *p* = 0.024). Detailed data are presented in Table 3. Index ablation and repeat ablation performed comparably in MACE (0.1% vs 0.0%, *p* = 1.0), MACCE (0.3% vs 0%, *p* = 0.23) and composite safety endpoint (1.2% vs 0.5%, *p* = 0.12). In-hospital recurrence of arrhythmia was significantly higher after index ablation compared to repeat procedure (7.4% vs. 4.5%, *p* = 0.004).

**Table 3:** Procedural-related complications during hospital stay

	Index	Repeat	*p* value
Severe complications	1.2% (41/3373)	0.5% (4/738)	0.12
Myocardial infarction	0.1% (2/3373)	0.0% (0/738)	1.00
Stroke	0.2% (7/3373)	0.0% (0/738)	0.36
Major bleeding requiring intervention	0.9% (32/3374)	0.5% (4/738)	0.38
Moderate complications	3.2% (95/2970)	2.2% (14/640)	0.20
Transient ischemic attack	0.1% (5/3373)	0.0% (0/738)	0.59
Cardiopulmonary resuscitation	0.0% (0/3373)	0.1% (1/737)	0.18
Aneurysmal hematoma. AV fistula	1.1% (38/3376)	1.1% (8/738)	1.00
Infection of puncture site	0.0% (1/2969)	0.0% (0/641)	1.00
Pericardial effusion	1.1% (33/2969)	0.5% (3/641)	0.19
Persistent AV block	0.0% (0/2969)	0.2% (1/641)	0.18
AV block III	0.1% (2/2969)	0.0% (0/641)	1.00
Sepsis	0.0% (0/2969)	0.0% (0/641)	
Endocarditis	0.0% (0/2969)	0.0% (0/641)	
Pulmonary embolism	0.0% (1/2969)	0.0% (0/641)	1.00
Pneumothorax	0.2% (7/2969)	0.0% (0/641)	0.62
Hemothorax	0.1% (4/2969)	0.0% (0/641)	1.00
Emergent cardiac surgery	0.1% (3/2969)	0.0% (0/641)	1.00
Phrenic nerve palsy	0.5% (15/3013)	0.0% (0/643)	0.089
Pulmonary vein stenosis	0.1% (2/2969)	0.2% (1/641)	0.44
Atrio-esophageal fistula	0.0% (0/2969)	0.0% (0/641)	
Minor complications	3.8% (113/2978)	2.0% (13/641)	0.024
Minor bleeding	3.3% (112/3373)	1.6% (12/737)	0.012
New AV block I° or II°	0.0% (1/2969)	0.2% (1/641)	0.32
New Right or left bundle branch block	0.0% (0/2969)	0.0% (0/641)	

All values given as percentages with total numbers in brackets. *P* values were calculated by Fisher’s exact test and considered significant if < 0.05.*

### Long-term follow-up: complication and safety

**Fig. 1:**
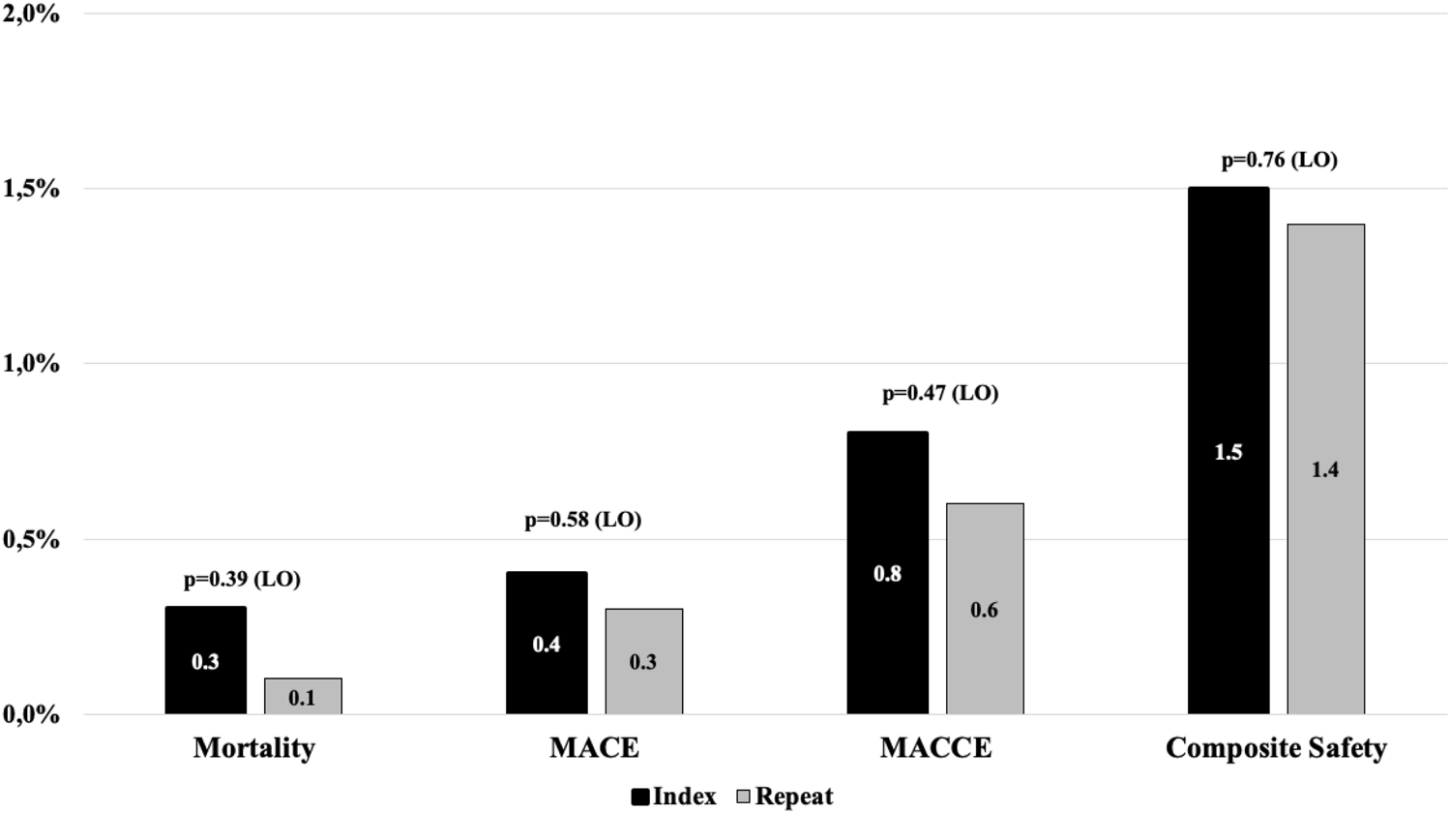
366-day safety follow-up by Kaplan–Meier method. MACE: composite endpoint of death and myocardial infarction; MACCE: composite endpoint of death, myocardial infarction, and stroke; Composite Safety Endpoint; composite endpoint of death, myocardial infarction, stroke, and major bleeding. *P* value < 0.05 was considered significant and calculated log-rank test

Follow-up information was obtained for 3304 patients (97.9%) in group I after a median of 457 days post-hospital discharge and for 718 patients (97.3%) in group II after a median of 463 days. There was no statistical difference between the groups in terms of mortality (0.3% vs 0.1%, *p* = 0.39), MACE (0.4% vs 0.3%, *p* = 0.58), MACCE (0.8% vs 0.6%, *p* = 0.47), and composite safety endpoint (1.5% vs 1.4%, *p* = 0.76). Both groups were comparable with regard to severe non-fatal AE (1.6% vs 2.4%, *p* = 0.20) and moderate non-fatal AE (8.3% vs 7.6%, *p* = 0.60). Incidence of PV stenosis (0.1% vs 0.0%, *p* = 0.35), phrenic nerve injury (0.5% vs 0.3%, *p* = 0.45), and atrio-esophageal fistula (0% vs 0.1%, *p* = 0.24) were not significantly different. Incidence of new cardiac device implantation was similar in both groups (2.5% vs 3.0%, *p* = 0.45). In Fig. 1, a graph with the 366-day safety data is given.

### Long-term follow-up: symptoms, recurrence, and satisfaction

**Fig. 2:**
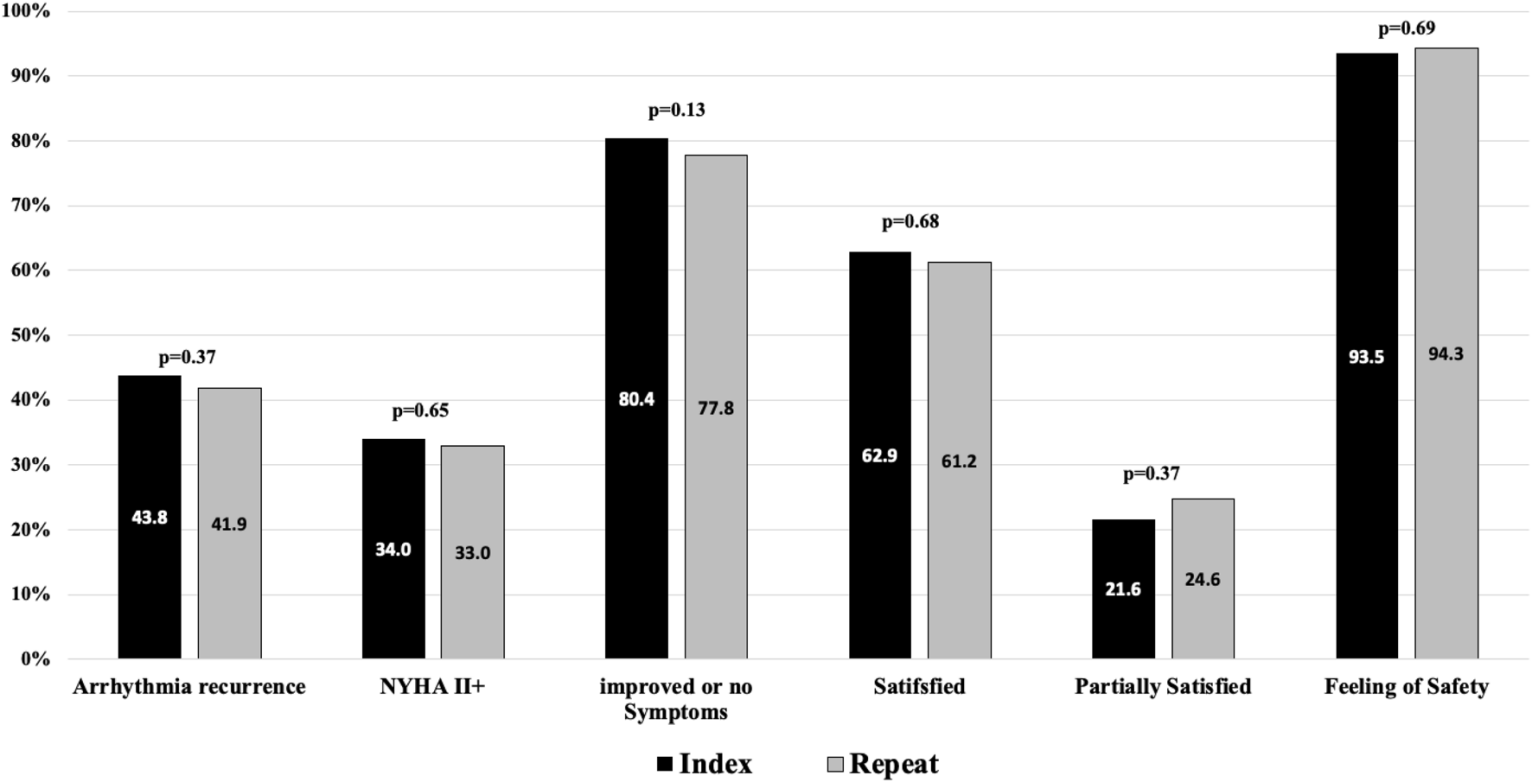
Follow-up of Arrhythmia Recurrence, Symptoms, and Patient-oriented outcomes. NYHA: New York Heart Association. *P* value < 0.05 was considered significant

**Fig. 3:**
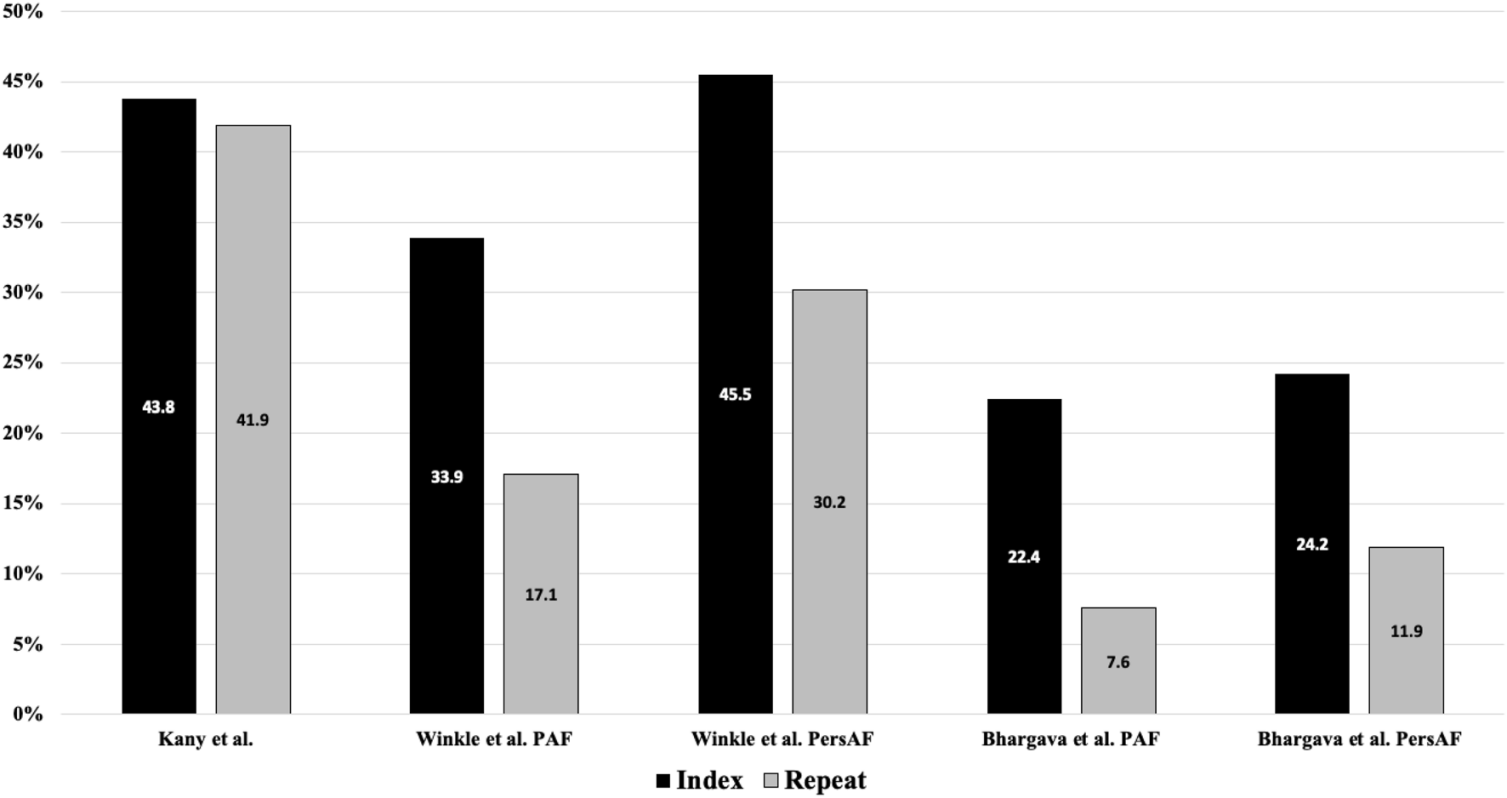
Comparison of arrhythmia recurrence to other index vs repeat catheter ablation cohorts. Respective works (Winkle et al. [25] and Bhargava et al. [26]) showing arrhythmia recurrence for paroxysmal atrial fibrillation (PAF) and persistent atrial fibrillation (PersAF)

Compared to the index cohort, patients after repeat ablation had comparable symptoms such as dyspnea and less, albeit not significantly, angina (16% vs 11.9%, *p* = 0.052). Both groups reported to have improved or no symptoms at high ratios (80.4% vs 77.8%, *p* = 0.13). There was no difference in the number of hospitalisations. Patients after repeat ablation were similarly satisfied or partially satisfied with the given treatment compared to the index group. In both groups, the number of unsatisfied patients was statistically similar. In both groups, the feeling of safety during treatment was equally high. Patient-oriented outcomes are visualized in Fig. 2. Arrhythmia recurrence rates were not significantly different in both groups. A graphical comparison to other index vs repeat catheter ablation studies is given in Fig. 3.

### Medical therapy at discharge

In both groups, antiarrhythmic therapy at discharge was similar in both groups (Table 4). Therapy with class I AAD (32.6% vs 30.1%, *p* = 0.18) and class III AAD (21.4% vs 24.4%, *p* = 0.081) was common. The therapy was mostly unchanged after the procedure, and in only 2.6% vs 2.8% (*p* = 0.9) of the cases, class III AAD was added to existing medication. The antithrombotic regime was mostly comprised of vitamin K antagonists (89.6% vs 91%, *p* = 0.26) which were mostly bridged by unfractionated heparin (8.3% vs 3.9%, *p* < 0.001) or low-molecular-weight heparin (63.3% vs 70.3%, *p* < 0.001).

**Table 4:** Medical therapy at discharge

Variable	Index (*n* = 3372)	Repeat (*n* = 735)	*p* value
Anti-arrhythmic drug (AAD) therapy			
Beta-blocker	74.5%	70.2%	0.017
AAD class I	32.6% (1099/3372)	30.1% (221/735)	0.18
AAD class III	21.4% (722/3372)	24.4% (179/735)	0.081
Sotalol	18.3% (132/722)	18.4% (33/179)	0.96
Amiodarone	79.2% (572/722)	78.8% (141/179)	0.89
AAD class III (added after procedure)	2.6% (19/722)	2.8% (5/179)	0.90
AAD class IV	1.6% (53/3372)	3.4% (25/735)	< 0.001
Antithrombotic therapy			
ASA	9.0% (304/3372)	6.7% (49/735)	0.040
Clopidogrel	2.7% (91/3372)	2.0% (15/735)	0.31
Vitamin K antagonist	89.6% (3023/3373)	91.0% (669/735)	0.26
Heparin (UFH)	8.3% (280/3372)	3.9% (29/735)	< 0.001
Heparin (LMWH)	63.3% (2136/3372)	70.3% (517/735)	< 0.001

*ASA* acetylsalicylic acid; *UFH* unfractionated heparin; *LMWH* low-molecular-weight heparin; All values given as percentages with total numbers in brackets. *P* values were calculated by Fisher’s exact test and considered significant if < 0.05.*

## Discussion

In this large, prospective multi-center cohort, we demonstrate that index as well as repeat ablation in AF are safe and provide significant improvement in symptoms and patients’ satisfaction. Additionally, these data demonstrated more extensive ablation strategies in repeat ablation with shorter EP study and fluoroscopy time as compared to index ablation.

Despite adequate rate control, symptomatic AF patients are often in need for rhythm control strategies such as PVI to reduce symptoms and improve quality of life. The need for the establishment of patient-oriented outcomes in clinical trials has been reiterated [9, 10].

However, early studies comparing rhythm control strategies in AF such as RACE and SAFE-T excluded patients with symptoms akin to NYHA class III or IV [11, 12]. In our study, we showed that a significant portion of patients (39.7%) undergoing index ablation reports symptoms of NYHA II or greater. In the cohort undergoing repeat ablation, almost half (49.4%) reported this severe symptomatic burden. Comparatively, in the recent CABANA trial, only 34.5% (in the ablation group) and 34% in the medical therapy group reported symptoms of ≥ NYHA II [13]. This may suggest that our real-world cohort undergoing AF ablation is more symptomatic than the collectives enrolled in large trials. In a sub-analysis of the CABANA trial, it was shown that AF ablation and anti-arrhythmic drug therapy are effective in reducing symptomatic burden with the former having significantly higher benefits as defined by the Atrial Fibrillation Effect on Quality of Life (AFEQT) Score (adjusted difference, 5.3 points [95% CI 3.7–6.9]; *P* < 0.001) [14]. There are, however, no data comparing the significant amount of repeat ablation to index procedures regarding procedure-related outcomes. We show that patients report equally high rates of improved or no symptoms after index ablation as well as after repeat ablation (80.4% vs 77.8%). However, both groups reported significant incidence of ≥ NYHA II symptoms 1 year after index (34%) or repeat (33%) ablation. Compared to baseline, our data show that repeat ablation reduces the number of patients experiencing severe symptoms. Considering that current guidelines emphasize patient choice for treatment indications, patient satisfaction is an increasingly important procedure-related outcome [15]. Both patient cohorts undergoing index or repeat ablation report being either fully satisfied (62.9% vs 61.2%) or partially satisfied (21.6% vs 24.7%). These data are comparable to other reported high incidences of patient satisfaction after AF ablation [7, 16]. Furthermore, less female patients in the repeat group suggest that women are less likely to be offered repeat ablation. An earlier analysis of the German Ablation Registry cohort showed that women were, in fact, more likely to have AF recurrence and more complications after catheter ablation [17].

PVI remains the only ablation strategy in AF with a proven effect on freedom of arrhythmia in long-term outcome. In our cohort, the repeat group was treated with additional lesions or CFAE significantly more often than the index group. However, the repeat group also had a higher number of patients with persistent AF (30.7% vs 38.1%). Catheter ablation in persistent AF (PersAF) and long-standing persistent AF is significantly less successful in maintaining SR than in PAF [18]. The STAR-AF II trial showed no benefit of additional liner lesions or CFAE with PVI in PersAF [19]. The data studied in this investigation include procedures done before the publication of STAR-AF II and has therefore to be seen in that context. The FIRE and ICE Redo study reported that 15% of patients undergoing index ablation are undergoing repeat ablation within a single year, comparable to the 20% described before [4, 20]. Ganesan et al. showed an average of 1.51 ablation procedures in a metanalysis of long-term outcome after ablation [18]. Therefore, safety in repeat procedures is of utmost importance. This study shows that in repeat ablation, despite additional lesions, EP study duration, fluoroscopy duration, and dose area product are significantly lower compared to index ablation. This finding is explained by the need for new circumferential wide area ablation at index ablation versus focused re-isolation in repeat cases. Consequently, less RF application time was noted in repeat ablation. This is in line with more recent data on repeat ablations from the CIRCA-DOSE trial [21]. Compared to other studies with similar comparisons, recurrence rates were higher in our study and generally in line with data from CIRCA-DOSE [3].

Furthermore, short-term complication or long-term MACE, MACCE, or composite safety were not different between repeat and index ablation. In line with existing data, we show that repeat ablation is safe in regard to procedural details, short- or long-term safety profile in a large, real-world cohort [6, 20, 22].

## Limitations

The German Ablation Registry gives insights into a large, prospective real-world cohort undergoing catheter ablation. Yet, there are some limitations. First, the procedures were carried out between 2007 and 2010, and technical aspects have, at least in part, been improved in AF ablation [23]. Major advancements include contact force catheters, new EAM systems, new RF protocols like high-power-short-duration, or new generations of cryoballoon devices [4, 24]. Another important difference is the change of periprocedural anticoagulation as uninterrupted regimes are favored currently compared with 2010.

Second, in a registry-based cohort, important confounding factors have to be considered and may not be known. Patients undergoing index ablation are not necessarily the same patients in the repeat ablation group. Therefore, the characteristics of the repeat group might be influenced by a selection bias (patients with persistent AF are more likely to receive a repeat ablation). Follow-up was also only centralized at the 1 year by IHF and left to each participating center before that. This was usually done in clinical routine and therefore not standardized (24 h vs 72 h Holter monitoring) and recurrence rates are therefore likely underestimated.

Complications and follow-up were also based on voluntary reports by health care workers and patients, a reporting bias has to be considered. Procedures and post-procedure care were dependent on each institute’s local standards and are therefore heterogenous in nature.

## Conclusion

Repeat ablation may offer symptomatic relief in patients with atrial fibrillation with high safety. Despite progressive forms of AF and more severe symptoms, the effects seem to be comparable to index ablation.

## Conflicts of interest

SK: none; JS and MH: the long-term follow-up and a prior publication were partially supported by unrestricted grants from Medtronic, Biosense Webster, and Biotronik; LE: received research support from several drug and device companies active in the field of electrophysiolopgy and received honoraria from several such companies in the past; D.T.: receiving lecture fees/honoraria from Bayer Vital, Boehringer Ingelheim Pharma, Bristol-Myers Squibb, Daiichi Sankyo, Medtronic, Pfizer Pharma, Sanofi-Aventis, St. Jude Medical and ZOLL CMS; LT.: lecture fees/honoraria from Medtronic, Abbott, Boston Scientific, Biotronik; AR received travel grants and lecture fees from Biosense Webster, Medtronic, Ablamap, Böhringer Ingelheim, Cardiofocus, EPD/Philips.

## Ethical approval

Ethics committee of the Rhineland‐Palatinate State Medical Council (Landesärztekammer Rheinland‐Pfalz) approved the study (No. 837.026.07 (5561)).

## Consent for publication

Given by all co-authors.

## Data Availability

All data and materials available at IFH Ludwigshafen.
